# Canine IVM With SOF Medium, Insulin-Transferrin-Selenium, and Low O_2_ Tension Improves Oocyte Meiotic Competence and Decreases Reactive Oxygen Species Levels

**DOI:** 10.3389/fcell.2021.694889

**Published:** 2021-09-07

**Authors:** Matteo Duque Rodriguez, Camila O. Cittadini, Gabriela M. Teplitz, Adrian De Stefano, Daniel M. Lombardo, Daniel F. Salamone

**Affiliations:** ^1^Facultad de Ciencias Agrarias, Politécnico Colombiano Jaime Isaza Cadavid, Medellin, Colombia; ^2^Laboratorio Biotecnología Animal, Departamento de Producción Animal, Facultad de Agronomía, Universidad de Buenos Aires, Buenos Aires, Argentina; ^3^Instituto de Investigaciones en Producción Animal, Universidad de Buenos Aires, Buenos Aires, Argentina; ^4^Facultad de Ciencias Veterinarias, Instituto de Investigación y Tecnología en Reproducción Animal, Catedra de Histología y Embriología, CONICET-Universidad de Buenos Aires, Buenos Aires, Argentina

**Keywords:** *in vitro* maturation, canine, oxygen tension, insulin-transferrin-selenium, SOF medium

## Abstract

Assisted reproductive technologies in canine species are limited due to the low efficiency of *in vitro* maturation (IVM). Unlike other mammals, bitches ovulate oocytes in the germinal vesicle stage and complete metaphase II (MII) after 48–72 h in the oviductal environment and become fertilizable. For this reason, we compared two different IVM media, synthetic oviductal fluid (SOF) supplemented with 8% bovine serum albumin (BSA) or a mixture of 8% BSA–2.5% fetal bovine serum (FBS) and TCM-199 with 10% FBS. Additionally, we evaluated the effect of supplementation with insulin-transferrin-selenium (ITS) and low O_2_ tension in oocyte maturation, reactive oxygen species (ROS) levels, membrane integrity, and embryo development following parthenogenetic activation (PA). After 72 h of culture, SOF + BSA, SOF + BSA + FBS, and TCM-199 + FBS show 5, 7, and 4% of MII, respectively, without a statistical difference. However, SOF + BSA produced significantly higher degeneration rates compared to SOF + BSA + FBS (44 and 23%, respectively). Remarkably, supplementation with 1 μl/ml of ITS under high O_2_ tension demonstrated a beneficial effect by improving maturation rates up to 20% compared to the other groups. Low O_2_ tension increased maturation rates to 36.5%, although there were no statistical differences compared to high O_2_ tension in the presence of ITS. Lower ROS levels and higher integrity of the cytoplasmic membrane were found in the presence of ITS despite no differences in maturation rates under low O_2_ tension groups. Additionally, after PA, 1% development until the eight-cell stage was obtained after activation of *in vitro*-matured oocytes in the presence of ITS. Taken together, these results indicate that SOF supplemented with 8% BSA and 2.5% FBS is suitable for IVM of canine oocytes and ITS supplementation was beneficial for both high and low O_2_ tension. Furthermore, the addition of ITS in the cultured system lowers ROS levels and increases membrane integrity in domestic dog oocytes after IVM.

## Highlights

– Insulin-transferrin-selenium supplementation in high O_2_ tension improves up to 20% MII of canine oocyte IVM from bitches of unknown age, breed, and stage of the estrous cycle.
– Low O_2_ tension increased maturation rates to 36.5%, although there were no statistical differences compared to high O_2_ with ITS.
– Higher integrity of the cytoplasmic membrane and lower reactive oxygen species (ROS) levels were found when COCs were cultured in the presence of ITS and low O_2_ atmosphere.

## Introduction

The low efficiency of *in vitro* maturation (IVM) in canine remains a real challenge for reproductive biologists (Van Soom et al., 2014). There are several aspects of the bitch’s reproductive physiology that differ from that of other domestic females. The bitch ovulates an immature oocyte [germinal vesicle (GV)] that corresponds to the prophase of the first meiotic division. The germinal vesicle breakdown (GVBD) occurs in the oviduct under high progesterone concentrations at 48–96 h after ovulation (Luvoni et al., 2005; Reynaud et al., 2012). Canine oocytes reach 10% of metaphase II (MII) after IVM, while most oocytes (30–40%) degenerate (Farstad, 2000; Luvoni, 2000). Some authors such as No et al. (2018) have used coculture with oviduct epithelial cells to improve maturation rates and have achieved 13% of MII using oocytes recovered from bitches at diestrus and 47% of MII with bitches at estrus. Most reports on canine IVM have been focused on the supplementation of the medium with hormones, such as estrogen, progesterone, follicle-stimulating hormone (FSH), luteinizing hormone (LH), human chorionic gonadotropin (HCG), and L-carnitine (Moawad et al., 2021). The medium TCM-199 is the medium of choice for IVM of most domestic species. However, synthetic oviductal fluid (SOF) was formulated based on the sheep oviductal fluid composition (Tervit et al., 1972), and it is interesting to evaluate due to the different location of the oocyte maturation in the dog. Previous authors have reported the use of SOF media for canine IVM with different supplementation and results (Hewitt and England, 1999; Bolamba et al., 2002; Saikhun et al., 2008; Lange-Consiglio et al., 2017).

Another component of the medium that is still under discussion is supplementation with fetal bovine serum (FBS) or bovine serum albumin (BSA). Some studies suggested that the use of FBS is beneficial during canine IVM due to its vitamins, hormones, and growth factors that it contains (Eppig and Schroeder, 1986; Luvoni et al., 2005; Rota and Cabianca, 2005). Serum is also capable of neutralizing the effect of potential toxic substances such as reactive oxygen species (ROS) (Rodrigues and Rodrigues, 2006). BSA, on the other hand, is capable of reducing toxic metabolites and ROS while promoting the uptake of other components like steroids, vitamins, fatty acids, and cholesterol (Bavister, 1995). Oviductal secretions include enzymatic defenses against ROS as a classic catalase and the family of glutathione peroxidase (Lapointe and Bilodeau, 2003; Saint-Dizier et al., 2020). The major antioxidants that control *in vivo* ROS levels in the oviduct remain to be characterized in species like the dog (Lapointe and Bilodeau, 2003). Furthermore, oxidative stress and ROS affect canine oocytes probably due to the high concentration of lipid droplets in the ooplasm (Tesoriero, 1981). Insulin-transferrin-selenium (ITS) has been used in IVM media of several species, generating positive outcomes (Jeong et al., 2008; Guimarães et al., 2016). While insulin promotes the absorption of nutrients, transferrin, and selenium have antioxidant properties (Jeong et al., 2008).

The main objective of the present study was to compare two different IVM media, one based on oviductal composition (SOF) supplemented with 8% BSA or a mixture of 8% BSA–2.5% FBS and a TCM-199 + 10% FBS. Additionally, we evaluated the supplementation of ITS and lowering O_2_ tensions.

## Materials and Methods

### Reagents

Except otherwise indicated, all chemicals were obtained from Sigma Chemical Company (St. Louis, MO, United States). Medium was prepared weekly and filtered through 0.22-mm pores (#4192 Acrodisc; Pall Corp., Ann Arbor, MI, United States) into sterile tubes.

### Experimental Design

#### Experiment 1: Media and Protein Supplementation Effect on Canine *in vitro* Maturation

We compared the ability of two different media to resume meiosis and reach MII stage of immature oocytes recovered after ovariectomy of bitches with unknown stage of the estrous cycle. The first medium consisted of TCM-199 supplemented with 10% v/v FBS (TCM-199 + FBS), and the second medium consisted of SOF supplemented with two different protein sources: 8% v/v BSA (SOF + BSA) or a mixture of 8% v/v BSA and 2.5% v/v FBS (SOF + BSA + FBS) based on our SOF media formulation (Buemo et al., 2016). For IVM, cumulus–oocyte complexes (COCs) were incubated in each maturation medium in an independent assay for 72 h (*n* = 237 COCs) and then 48 h (*n* = 96 COCs) on high O_2_ tension in humidified air at 38.5°C. Three biological replicates were performed for this experiment.

#### Experiment 2: Insulin-Transferrin-Selenium Effects on Canine *in vitro* Maturation

The optimal medium determined in the first experiment (SOF + 8% BSA + 2.5% FBS) was supplemented with two concentrations of ITS. SOF + BSA + FBS in the absence of ITS was used as control. Maturation medium was supplemented with 1 and 10 μl/ml of ITS (41400045; Thermo Fisher Scientific; insulin: 0.1721763 mM, transferrin: 0.006875 mM, and selenium: 0.0038728325 mM). For IVM, COCs (*n* = 232) were incubated in each maturation medium for 72 h on high O_2_ tension in humidified air at 38.5°C. Three biological replicates were performed for this experiment.

#### Experiment 3: The Effect of O_2_ Tension and Insulin-Transferrin-Selenium Supplementation on Canine *in vitro* Maturation

To evaluate the effect of high (20%) and low (5%) O_2_ tension and ITS supplementation, COCs (*n* = 245) were incubated in SOF + 8% BSA + 2.5% FBS in the presence (1 μl/ml) or absence of ITS in a controlled atmosphere with high or low O_2_ tension. It was decided to perform IVM for 48 h instead of 72 h based on the optimum maturation time assessed in experiment 1. Four groups were selected: (A) SOF + BSA + FBS + high O_2_; (B) SOF + BSA + FBS + 1 μl/ml of ITS + high O_2_; (C) SOF + BSA + FBS + low O_2_; and (D) SOF + BSA + FBS + 1 μl/ml of ITS + low O_2_. After maturation with low O_2_ tension in the presence or absence of ITS, oocytes were used to analyze ROS levels (*n* = 72), membrane integrity (*n* = 138), and parthenogenetic activation (PA) (*n* = 163) through dichlorodihydrofluorescein diacetate (DCHFDA) co-stained with propidium iodide (PI) and ionomycin and 6-dimethylaminopurine (6-DMAP) for activation. Three biological replicates were performed for this experiment.

### Cumulus–Oocyte Complexes Collection and *in vitro* Maturation

**FIGURE 1:**
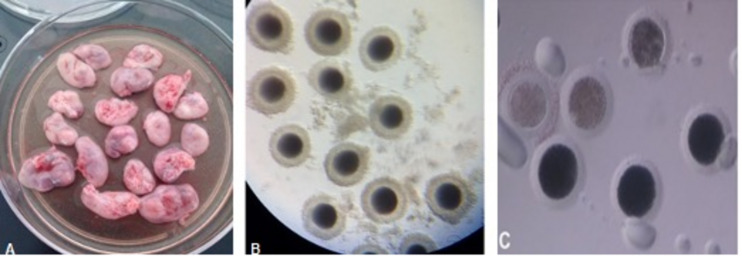
**(A)** Bitch ovaries that were previously washed and freed from ovarian tissue. **(B)** Grade I cumulus–oocyte complexes (COCs) recovered from the ovaries by the slicing method. The COCs had dark and uniform cytoplasm, large size, and two or more layers of cumulus cells. **(C)** Denuded canine oocytes. The presence of pale-looking oocytes is observed after 72 h of *in vitro* maturation (IVM).

In this study, a total of 197 ovaries were used from bitches of unknown age, breed, and stage of the estrous cycle. The ovaries were obtained from the Tigre Zoonotic Center (Tigre, Buenos Aires, Argentina) after routine ovariectomy. Ovaries were transported to the laboratory in Falcon tubes containing 10 ml of 4-(2-hydroxyethyl)-1-piperazineethanesulfonic acid (HEPES)-buffered Tyrode’s medium containing albumin, lactate, and pyruvate [TALP-HEPES (Bavister and Yanagimachi, 1977)] at 20–25°C and were processed within 6 h after extraction. Once in the laboratory, the ovaries were extracted from the ovarian bursa and washed in TALP-HEPES medium at 38.5°C. Clean ovaries were processed as follows. First, a longitudinal cut with a scalpel followed by numerous punctures with needle and scraping of the ovarian surface was performed to release the COCs to the medium. COCs were selected based on previously established morphological criteria (Hewitt and England, 1999). Briefly, COCs that had a larger size, were surrounded by more than two layers of cumulus cells, had dark and uniform cytoplasm were selected and categorized as grade I (Figure 1). Oocytes that were small, fragmented or had a pale cytoplasm were discarded. For experiment 1, the first medium consisted of TCM-199 (31100-035; Thermo Fisher Scientific, Waltham, MA, United States) supplemented with 2.5 mM sodium pyruvate (P2256), 0.57 mM L-cysteine (C7352), 10 μg/ml FSH (NIH-FSH-P1; Folltropin, Athens, Georgia, United States), 10 ng/ml epidermal growth factor (EGF) (PHG0314, Thermo Fisher Scientific), 1% v/v antibiotic (15240-096; Thermo Fisher Scientific), and 10% v/v FBS (013/07; Internegocios S.A., Mercedes, Buenos Aires, Argentina). The second medium consisted of SOF (Tervit et al., 1972) supplemented with 10 μg/ml FSH, 10 ng/ml EGF, and two different protein sources: 8% BSA (A6003) or a mixture of 8% BSA and 2.5% FBS. Groups of up to 20 grade I COCs were incubated in 100-μl droplets of the selected medium, coated with mineral oil (M8410). Plates were incubated under high O_2_ (5% CO_2_) tension and humidified air at 38.5°C for 48 or 72 h (depending on the experiment). The medium was renewed every 24 h.

### Evaluation of Nuclear Stage

**FIGURE 2:**
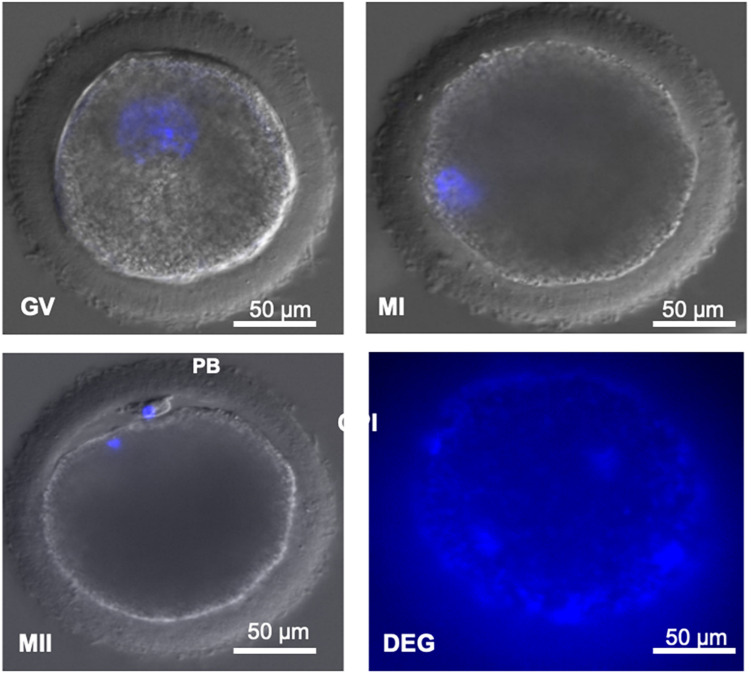
(GV) Decondensed DNA contained in a large germinal vesicle. (MI) Condensed DNA. (MII) Condensed DNA and presence of the first polar body. (PB) First polar body. Degenerated (DEG) oocytes without identifiable material. Image obtained under confocal microscope (magnification ×400). Scale bar: 50 μm.

After maturation, for nuclear stage assessment, oocytes were first denuded by repeated passages through a glass pipette with an appropriate diameter. Once denuded, oocytes were permeabilized by incubation in 0.3% Triton-X (T-9284) for 15 min and washed in TALP-HEPES. Following permeabilization, oocytes were incubated in 1% v/v Hoechst 33342 (B-2261) droplets for 20 min in darkness at room temperature. Oocytes were washed three times in TALP-HEPES and mounted on glass slides for observation under UV light (excitation and emission wavelengths of 350 and 461 nm, respectively). The glass slides were covered with a coverslip supported by four small droplets of paraffin in the corner in order to regulate the distance between both glasses without breaking the oocytes. Nuclear stage assessment was scanned using an inverted confocal microscope (Olympus IX83 Spinning Disk Confocal System). Oocytes were classified based on the morphology of the nucleus as shown in Figure 2 and based on previously established criteria (Hewitt and England, 1999).

### Assessment of Reactive Oxygen Species Levels

Reactive oxygen species levels were measured by DCHFDA staining as described by Lorenzo et al. (2019). For ROS measurement by the DCHFDA method, COCs (*n* = 72) were denuded as previously described. Oocytes were incubated with 153 μM of DCHFDA (D6883) for 30 min in darkness at 37°C. Oocytes were mounted on glass slides for observation under an epifluorescent microscope (Leica DM4000B LED trinocular connected to a Leica DCC-380X camera). Images were captured using LAS version 4.2.0 microscope software (Leica) using excitation and emission wavelengths of 450–490 and 515–565 nm, respectively. The greater the intensity of green fluorescence, the greater the amount of ROS. Fluorescence intensity was determined by the transmittance variable measured by digital morphometry on images captured using Leica Qwin V3.1 software. The transmittance arbitrary units corresponded to levels between 0 and 256 for each of the Red–Green–Blue (RGB) channels for the pixels of the analyzed matrix. The mean transmittance of each experimental group was recorded and compared. ROS levels were measured in oocytes matured with SOF + BSA + FBS medium in the presence (1 μl/ml) or absence of ITS under low O_2_ (5% CO_2_, 5% O_2_, and 90% N_2_) tension in humidified air at 38.5°C for 48 h. Three biological replicates were performed.

### Viability Assessment

Aside from the ROS level assessment, oocytes from the two groups described in the previous section were co-stained with PI in order to evaluate oocyte viability and membrane integrity. Briefly, COCs (*n* = 138) matured in SOF + BSA + FBS medium in the presence (1 μl/ml) or absence of ITS under low O_2_ tension in humidified air at 38.5°C for 48 h were assessed. For co-staining, oocytes were incubated in a 1% v/v PI (P4170) solution for 5 min at 37°C. Oocytes were washed and placed between a glass slide with a coverslip and examined under epifluorescence microscope using excitation and emission wavelengths of 450–490 and 515–565 nm, respectively. Three biological replicates were performed.

### Parthenogenetic Activation

Oocytes (*n* = 163) that had been previously exposed to the most optimal maturation conditions (SOF + BSA + FBS under low O_2_ tension) for 48 h in the presence (1 μl/ml) or absence of ITS were chemically activated. After IVM, oocytes were denuded and subjected to activation with ionomycin (I24222; Thermo Fisher Scientific) and 6-DMAP (D2629). Briefly, oocytes were incubated for 4 min in TALP-HEPES with 1 μl/ml ionomycin and then washed three times. Oocytes were then incubated in a 1% v/v 6-DMAP solution in SOF medium supplemented with 2.5% FBS for 4 h. Oocytes were then washed thoroughly in TALP-HEPES and cultured. Oocyte culture was performed in SOF droplets supplemented with 2.5% FBS (without hormones) in low O_2_ tension in humidified air at 38.5°C. Seven days after activation, oocytes were evaluated for cleavage. Three biological replicates were performed.

### Statistical Analysis

All data were analyzed by the Chi-square test using GraphPad Prism 6 software. To compare ROS levels between groups, an unpaired two-tailed *t*-test was performed, and to assess viability, a comparison of proportions (Fisher test) was performed using GraphPad Prism 6 software. Statistical significance was determined when the *p*-value was <0.05.

## Results

The experiments reported in this study were carried out over a period of 1 year. During this year, a total of 1,305 COCs were collected from bitches of unknown age, breed, and stage of the estrous cycle. It was possible to obtain information on the recovery rates per ovary during the different seasons of the year (Table 1). We found that the recovery rate of COCs grade I per ovary was 8.6 ± 1.7 in fall, 8.9 ± 2.1 in winter, 9.1 ± 1.1 in summer, and 6.3 ± 1.4 in spring. No significant differences were found between the different seasons.

**TABLE 1:** Grade I COCs collected per ovary during different seasons of the year.

Season	No. ovaries	No. COCs grade I	Recovery rate per ovary
Spring	72	452	6.3 ± 1.4
Summer	18	160	9.1 ± 1.13
Fall	53	473	8.6 ± 1.7
Winter	27	220	8.9 ± 2.1
Total	170	1,305	–

*In the same column, percentages without the same superscripts differed significantly (*p*-value < 0.05).*

*COC, cumulus–oocyte complex.*

### Experiment 1: Media and Protein Supplementation Effect on Canine *in vitro* Maturation

After 72 h of culture, the maturation rates (MII) were low for the three media, 4% for TCM-199 + FBS, 5% for SOF + BSA, and 7% for SOF + BSA + FBS without significant differences between them. Most of the oocytes were arrested at the metaphase I (MI) stage or did not resume meiosis. Degeneration rates were higher than 20% in all cases, but in SOF + BSA medium, it was significantly higher (44%, *p* ≤ 0.05; Table 2). For this reason, SOF + BSA was discarded for experiment 2. The rate of non-identifiable oocytes (NN) was higher in SOF + BSA + FBS, and this category corresponds to oocytes that could belong to any of the previous categories. Moreover, the presence of oocytes with unidentifiable nuclear material could be related to difficulties in the staining methods that is common in this species. The presence of oocytes with a pale or lightly pigmented cytoplasm after 72 h of culture is associated with degeneration (Figure 1). After 48 h of maturation in TCM-199 + FBS and SOF + BSA + FBS media, similar results to those of 72 h were found with no significant differences (Table 2). In addition, maturation rates did not change, supporting the hypothesis that a longer period of time during IVM contributes to degeneration of canine oocytes without improving maturation rates (Table 2).

**TABLE 2:** *In vitro* maturation of canine oocytes for 72 and 48 h in three different media under high O_2_ atmosphere.

Group	Time IVM	No. oocytes	GV	MI	MII	DEG	NN
TCM-199 + FBS	72	73	16 (21.9%)^a^	24 (32.8%)^a^	3 (4.1%)^a^	22 (30.1%)^*a,c*^	8 (10.9%)^a^
	48	41	8 (19.5%)^a^	21 (51.2%)^b^	4 (9.7%)^a^	3 (7.3%)^b^	5 (12.1%)^a^
SOF + BSA + FBS	72	86	17 (19.7%)^a^	23 (26.9%)^a^	6 (6.9%)^a^	20 (23.2%)^*c*^	20 (23.2%)^b^
	48	55	16 (29%)^a^	27 (49%)^b^	2 (3.6%)^a^	5 (9%)^b^	5 (9%)^a^
SOF + BSA	72	78	16 (20.5%)^a^	21 (26.9%)^a^	4 (5.5%)^a^	34 (43.5%)^a^	3 (4.1%)^a^

*The percentages of oocytes in the germinal vesicle (GV), metaphase I (MI), metaphase II (MII), degenerated (DEG), and non-identifiable (NN) stages were recorded. In the same column, percentages without the same superscripts differed significantly (^*a*−*c*^*p*-value < 0.05). The experiments were repeated three times (*n* = 3).*

*BSA, bovine serum albumin; FBS, fetal bovine serum; IVM, *in vitro* maturation; SOF, synthetic oviductal fluid.*

### Experiment 2: Insulin-Transferrin-Selenium Effect on Canine *in vitro* Maturation

In order to evaluate the effect of antioxidant supplementation on canine IVM, maturation was performed using SOF + BSA + FBS medium in the presence of different concentrations of ITS for 72 h. It was found that supplementation with 1 μl/ml ITS significantly improved canine IVM, increasing the proportion of oocytes that reached the MII stage (20%). No significant differences were observed in the proportion of oocytes arrested at the GV and MI stages or in the proportion of oocytes with NN between groups (Table 3). It was observed that the proportion of degenerated (DEG) oocytes was significantly higher in 10 μl/ml ITS.

**TABLE 3:** The effect of ITS supplementation on canine IVM for 72 h under high O_2_ atmosphere.

Group	No. oocytes	GV	MI	MII	DEG	NN
SOF + BSA + FBS	71	16 (22.5%)^a^	37 (52.1%)^a^	3 (4.2%)^a^	7 (9.8%)^a,b^	8 (11.2%)^a^
SOF + BSA + FBS + 1 μl/ml ITS	81	16 (19.7%)^a^	32 (39.5%)^a^	16 (19.7%)^b^	7 (8.6%)^a^	10 (12.3%)^a^
SOF + BSA + FBS + 10 μl/ml ITS	80	16 (20%)^a^	29 (36.2%)^a^	5 (6.2%)^a^	17 (21.2%)^b^	13 (16.2%)^a^

*SOF + BSA + FBS (control): COCs cultured in SOF + 8% BSA + 2.5% FBS; SOF + BSA + FBS + 1 μl/ml ITS: COCs cultured in SOF + 8% BSA + 2.5% FBS and 1 μl/ml ITS; and SOF + BSA + FBS + 10 μl/ml ITS: COCs cultured in SOF + 8% BSA + 2.5% FBS and 10 μl/ml ITS. The percentages of oocytes in the germinal vesicle (GV), metaphase I (MI), metaphase II (MII), degenerated (DEG), and non-identifiable (NN) stages was recorded. In the same column, percentages with different superscripts differed significantly (^*a*,*b*^*p* < 0.05). The experiments were repeated three times (*n* = 3).*

*BSA, bovine serum albumin; COC, cumulus–oocyte complex; FBS, fetal bovine serum; ITS, insulin-transferrin-selenium; IVM, *in vitro* maturation; SOF, synthetic oviductal fluid.*

### Experiment 3: The Effect of O_2_ Tension and Insulin-Transferrin-Selenium Supplementation on Canine *in vitro* Maturation

After 48 h of IVM, it was observed that the maturation rate of SOF + BSA + FBS + high O_2_ was significantly lower than those in the other groups (Table 4). It is important that excluding SOF + BSA + FBS + high O_2_, maturation rates exceeded 20% without significant differences between them. On the other hand, it was observed that the proportion of oocytes arrested at the MI stage was greater than 30% in all groups. The number of oocytes in the GV stage was also higher than those in groups that had been cultured under high O_2_ tension. These results indicate that low O_2_ tension either in the presence or absence of ITS was able to resume meiosis more successfully. Finally, no significant differences were observed in the rate of DEG oocytes for any group, but it is an interesting fact that the degeneration rates were less than 10% in all cases.

**TABLE 4:** *In vitro* maturation of canine oocytes in SOF + BSA + FBS medium for 48 h under two different O_2_ tension atmospheres in the presence or absence of ITS.

Group	No. oocytes	GV	MI	MII	DEG	NN
SOF + BSA + FBS + high O_2_	64	26 (40.6%)^a^	27 (42.2%)^a^	5 (7.8%)^a^	6 (9.4%)^a^	0 (0%)^a^
SOF + BSA + FBS + 1 μl/ml ITS + high O_2_	53	16 (30.2%)^*a,c*^	20 (37.7%)^a^	13 (24.5%)^b^	2 (3.8%)^a^	2 (3.8%)^a^
SOF + BSA + FBS + low O_2_	63	5 (7.9%)^b^	32 (50.8%)^a^	23 (36.5%)^b^	1 (1.6%)^a^	2 (3.2%)^a^
SOF + BSA + FBS + 1 μl/ml ITS + low O_2_	65	11 (16.9%)^*b,c*^	34 (52.3%)^a^	17 (26.2%)^b^	2 (3.1%)^a^	1 (1.5%)^a^

*The percentages of oocytes in the germinal vesicle (GV), metaphase I (MI), metaphase II (MII), degenerated (DEG), and non-identifiable (NN) stages were recorded. In the same column, percentages with different superscripts differed significantly (^*a*−*b*^*p* < 0.05). The experiments were repeated three times (*n* = 3).*

*BSA, bovine serum albumin; FBS, fetal bovine serum; ITS, insulin-transferrin-selenium; IVM, *in vitro* maturation; SOF, synthetic oviductal fluid.*

### Assessment of Reactive Oxygen Species Levels

**FIGURE 3:**
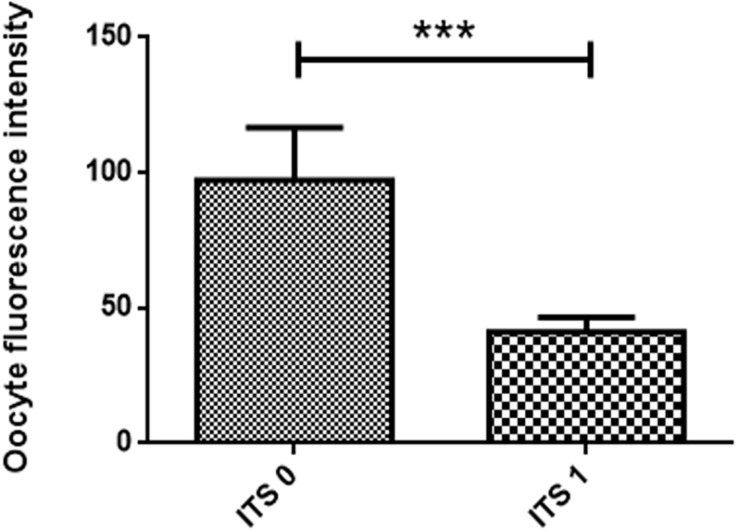
Effect of insulin-transferrin-selenium (ITS) on oocyte reactive oxygen species level. Data show mean ± SEM. Oocyte fluorescence intensity (transmittance) in each experimental group after staining with 2′,7′-dichlorodihydrofluorescein diacetate (three replicates). ITS 0: oocytes matured in the absence of ITS; ITS 1: oocytes matured in the presence of 1 μl/ml of ITS. The meaning of “***” is that the statistical difference is significant.

Reactive oxygen species levels were measured for the most suitable treatment groups determined in the previous experiments (SOF + BSA + FBS medium in the presence or absence of 1 μl/ml of ITS under low O_2_ tension). ROS levels were found to be significantly higher in the absence of ITS (97.23 ± 19.49; *n* = 31) compared to 1 μl/ml of ITS (41.19 ± 5.32; *n* = 42) as evidenced by oocyte fluorescence intensity measurement in Figure 3.

### Viability Assessment

Aside from the ROS level assessment, oocytes cultured in SOF + BSA + FBS medium in the presence (1 μl/ml) or absence of ITS under low O_2_ tension were co-stained with PI in order to evaluate maturation and cell membrane integrity. Oocyte viability was significantly higher in the ITS-supplemented group (56.3 vs. 23.8%; Table 5). In this experiment, oocytes matured in SOF + BSA + FBS + 1 μl/ml of ITS had significantly higher maturation rates (25.3%) compared to that in the absence of ITS (11.9%; Table 5).

**TABLE 5:** Viability and IVM of canine oocytes in SOF + BSA + FBS medium for 48 h under low O_2_ tension atmospheres in the presence or absence of ITS.

Group	No. oocytes	Viability	GV + MI	MII	NN
SOF + BSA + FBS + low O_2_	67	16 (23.8%)^a^	59 (88.1%)^a^	8 (11.9%)^a^	9 (13.4%)^a^
SOF + BSA + FBS + 1 μl/ml ITS + low O_2_	71	40 (56.3%)^b^	53 (74.6%)^a^	18 (25.3%)^b^	10 (14.1%)^a^

*Viability and IVM of oocyte after co-staining with propidium iodide (PI) and Hoechst 33342 to determine maturation and cell membrane integrity. The percentages of oocytes in the germinal vesicle or metaphase I (GV + MI), metaphase II (MII), and non-identifiable (NN) stages were recorded simultaneously with cell membrane integrity evaluation after 48 h of IVM. In the same column, percentages with different superscripts differed significantly (^*a*,*b*^*p* < 0.05). The experiments were repeated three times (*n* = 3).*

*BSA, bovine serum albumin; FBS, fetal bovine serum; IVM, *in vitro* maturation; ITS, insulin-transferrin-selenium; SOF, synthetic oviductal fluid.*

### Parthenogenetic Activation and Embryo Development

**FIGURE 4:**
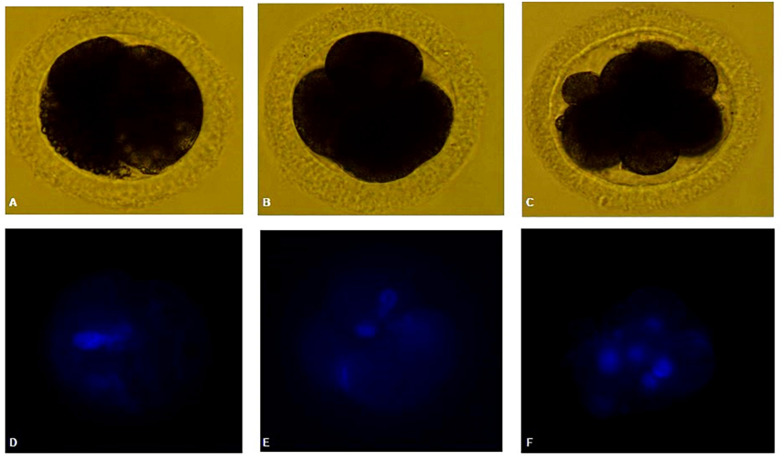
**(A,D)** Canine oocyte at 2-cell stage. **(B,E)** Canine oocyte at four-cell stage. **(C,F)** Canine oocyte at eight-cell stage. Parthenogenetic embryos visualized by bright field and Hoechst staining.

Parthenogenetic activation was performed after oocyte maturation in SOF + BSA + FBS medium in the presence (1 μl/ml) or absence of ITS under low O_2_ tension. After 7 days of culture, it was found that a high proportion of oocytes was DEG or had unidentifiable material (DEG + NN). This proportion was significantly higher in the group that had been matured in the absence of ITS compared to that in the 1-μl/ml ITS group (94.4 and 71.4%, respectively). Cleaved oocytes were obtained, being two oocytes from each group arrested at the 2- to 4-cell stage. A single oocyte from SOF + BSA + FBS + 1 μl/ml ITS + low O_2_ tension was found at a more advanced stage of development, arrested at the eight-cell stage (Figure 4). Neither morula nor blastocysts were obtained. The cleavage rates were very low in both groups (2.8 and 3.3%, respectively); however, the only oocyte in the eight-cell stage was obtained in the group supplemented with 1 μl/ml of ITS.

## Discussion

We evaluated different strategies to improve IVM, including media compositions, ITS supplementation, and low O_2_ tension. Most of the reproductive technologies that produced puppies as *in vitro* fertilization (IVF) and cloning were done using *in vivo*-matured oocytes, which has several welfare- and cost-associated problems (Jang et al., 2010; Oh et al., 2018). Therefore, improvement of IVM in the dog would allow the use of oocytes obtained from regular ovariectomies in veterinary clinics, which would favor the development of reproductive technologies. Although several reports have been made to improve canine IVM performance, results are still suboptimal with less than 10% of matured oocytes (Luvoni, 2006; Nagashima et al., 2019).

Experiment 1 compared different media and sources of protein supplementation. Maturation rates were low for all the treatment groups without differences, but lower degeneration rates were found in SOF + BSA + FBS compared with SOF + BSA. Similar to our findings, previous reports also compared TCM-199 with SOF medium, with similar MII rates (<10%) (Rota and Cabianca, 2005; Evecen et al., 2009). Additionally, Lange-Consiglio et al. (2017) reported that 2.5% FBS in SOF medium also had similar degeneration rates to our results. It is clear that independent of the media and supplementation, *in vitro* meiotic resumption of dog oocytes is low. For this reason, in the next experiments, we tried to identify some of the lacking or detrimental components of the culture system.

Dog oocytes contain a large amount of intracellular lipids that are likely to be susceptible to oxidative stress. Results demonstrated that 1 μl/ml of ITS in high O_2_ tension improved significantly the MII rates up to 20%, supporting the hypothesis that ITS is important for IVM of canine oocytes. The first report that used ITS in canine IVM using TCM-199 was Rota and Cabianca (2005), without significant improvement in MII rates with markedly low results (<3%). However, Saikhun et al. (2008), who compared different media using ITS in all of them, reported high MII rates in accordance with our results. Other authors showed in bovine and feline that ITS has a beneficial effect on embryo developmental competence (Moro et al., 2014; Guimarães et al., 2016) but not better maturation rates. Otherwise, in the dog, we determined that ITS under a high O_2_ culture system duplicated MII rates.

Oxygen concentrations in the oviduct are one-third of the atmosphere and, for that reason, IVM in experiment 3 was performed in two different oxygen tensions (Rodrigues and Rodrigues, 2010). Canine and pig oocytes have the highest amount of lipid droplets and also longer maturation periods, making them more prone to oxidative stress due to lipid peroxidation with potential damage to the cell membrane (Bradley and Swann, 2019; Dalbies-Tran et al., 2020). Therefore, it would be reasonable to assume that by reducing or alleviating the effect of oxidative stress by antioxidant supplementation in an oxygen-depleted atmosphere improves canine IVM. We showed that lower O_2_ tensions are beneficial for canine IVM, since higher maturation rates and lower degeneration rates were obtained when COCs were cultured in low O_2_ tension in either the presence or absence of ITS. Salavati et al. (2012) also compared high and low O_2_ tensions but with remarkably low results and without differences between treatments. These outcomes are in agreement with previous reports in other species that have shown beneficial effects of lowering O_2_ tension to physiological levels during IVM (Salavati et al., 2012).

The optimal medium SOF + BSA + FBS and low O_2_ tension in the presence or absence of ITS were further investigated for ROS levels, viability assay, and PA after 48 h of IVM. Although no differences in maturation rates were demonstrated, the presence of ITS and low O_2_ tension decreased ROS levels considerably (Figure 3) and increased oocytes with membrane integrity after maturation. Transferrin constitutes a major protein component in follicular and ampullary fluids and also acts as a chelator of highly toxic hydroxyl radicals to limit oxidative stress (Das et al., 2014). Selenium protects the cells from oxidative damage by reducing free radical production and inhibiting lipid peroxidation (Córdova et al., 2010). In addition, insulin is a key metabolic hormone influencing energy metabolism at different levels. It has been documented that insulin binds to its receptors in oocytes, granulosa cells, and both theca cells of healthy follicles, supporting the hypothesis that this hormone plays a role in oocyte maturation, follicular growth, and stroma cell function (Alvarez et al., 2019).

Finally, embryo development after PA was compared in the presence or absence of ITS supplementation under low O_2_ tensions, and cleavage rates were low for both groups, and there were no statistical differences between them. Only one oocyte belonging to the 1-μl/ml ITS group was found at the eight-cell stage, while oocytes at the 2- to 4-cell stage could be obtained for both groups (Figure 4). To our knowledge, this is the first report of ionomycin and 6-DMAP PA of *in vitro*-matured oocytes and developed up to eight-cell stage. Comparison of different activation protocols using *in vivo*-matured oocytes has been reported, reaching 25% of eight-cell stage embryos with no blastocyst obtained using ionomycin and 6-DMAP for PA (Jang et al., 2008).

## Conclusion

In summary, we could conclude that SOF with 8% BSA and 2.5% FBS is suitable for IVM, and ITS supplementation in high O_2_ tension improves up to 20% MII of canine oocytes from bitches of unknown age, breed, and stage of the estrous cycle. Low O_2_ tension increased maturation rates to 36.5%, although there were no statistical differences compared to high O_2_ tension in the presence of ITS. Additionally, higher integrity of the cytoplasmic membrane and lower ROS levels were found when COCs were cultured in the presence of ITS. These findings will help to improve *in vitro* embryo production and the efficiency of assisted reproduction in canine species.

## Data Availability Statement

The original contributions presented in the study are included in the article/supplementary material, further inquiries can be directed to the corresponding author.

## Author Contributions

MD and CC: conceptualization, methodology, formal analysis, and writing—original draft. GT, AD, and DL: methodology and formal analysis. DS: supervision, project administration, writing, review and editing, and funding acquisition. All authors contributed to the article and approved the submitted version.

## Conflict of Interest

The authors declare that the research was conducted in the absence of any commercial or financial relationships that could be construed as a potential conflict of interest.

## Publisher’s Note

All claims expressed in this article are solely those of the authors and do not necessarily represent those of their affiliated organizations, or those of the publisher, the editors and the reviewers. Any product that may be evaluated in this article, or claim that may be made by its manufacturer, is not guaranteed or endorsed by the publisher.
